# Should We Use Citizens' Assemblies to Make Health Policy?

**DOI:** 10.1111/bioe.70111

**Published:** 2026-04-21

**Authors:** Daniel Hutton Ferris, Johannes Kniess

**Affiliations:** ^1^ Politics, Newcastle University Newcastle upon Tyne UK

**Keywords:** accountability for reasonableness, citizen deliberation, citizens' assemblies, deliberative democracy, health policy, minipublics

## Abstract

This article assesses the normative case for using citizens' assemblies—small deliberative forums of randomly selected citizens—in health policymaking. Although they are increasingly popular, their normative justification remains underexplored. We reconstruct three possible rationales: Norman Daniels's ‘Accountability for Reasonableness’ (A4R) framework; an epistemic argument emphasising the value of outsider perspectives for making more just decisions; and a deliberative democratic argument focused on promoting legitimacy in the bureaucracy. We argue that A4R offers only weak and contingent support for citizens' assemblies. The epistemic argument highlights the value of lay perspectives in identifying epistemic blind spots but lacks clarity on when it outperforms expert knowledge. The deliberative democratic rationale is more compelling in potentially generating some kinds of legitimacy but applies only in a limited range of scenarios. We therefore conclude that the normative case for using citizens' assemblies is not as strong as their popularity in policymaking would lead us to believe.

## 1 Introduction

Health policy inevitably raises moral questions upon which there is considerable disagreement. Examples include the allocation of scarce healthcare resources, public health interventions that involve trade‐offs between freedom and safety, the regulation of novel technologies (such as genome editing) or contested approaches in research ethics. How can we make fair and legitimate policy choices in cases like these?

One increasingly popular answer is to involve ordinary citizens in deliberation. Indeed, according to the World Health Organization, ‘citizen engagement is a fundamental component of evidence‐informed policy in the health field’ [1, 2]. The engagement in question is typically not mass deliberation, but rather deliberation within small forums whose composition reflects society on a small scale: ‘citizens' assemblies.’ These forums bring together a representative sample of citizens to learn and deliberate about a topic and thereby inform policymaking^1^ [3]. Although calls for increased citizen participation have now become commonplace in bioethics [4], there has been relatively little in‐depth theoretical discussion about the use of citizens' assemblies in health policy.

This article addresses this gap by systematically examining the normative case for using citizens' assemblies in health policy. After introducing the idea of citizens' assemblies (Section 2), we proceed to reconstruct three possible normative rationales for their use: Norman Daniels's influential ‘Accountability for Reasonableness’ (A4R) framework (Section 3); an epistemic argument emphasising the value of outsider perspectives for making more just decisions (Section 4); and a deliberative democratic argument focused on promoting legitimacy in the bureaucracy (Section 5). We argue that A4R offers only weak and contingent support for citizens' assemblies. The epistemic argument highlights the value of lay perspectives in identifying epistemic blind spots but lacks clarity on when it outperforms expert knowledge. The deliberative democratic rationale is more compelling in its explanation of how citizens' assemblies might generate political legitimacy, but it applies only in a limited range of scenarios.

We therefore conclude that the normative case for using citizens' assemblies is not as strong as their popularity in policymaking would lead us to believe. In the final section, we explore alternative empirical explanations for their rise: officials may value citizens' assemblies less for their inherent potential to promote justice or normative legitimacy than for their strategic advantages or ability to generate empirical or *de facto* legitimacy in an era of declining political trust. Whatever the reasons, however, it is not clear that there are strong principled normative reasons to employ citizens' assemblies to resolve the dilemmas of health policymaking.

## 2 Citizens' Assemblies

Citizens' assemblies are participatory democratic innovations that have generated considerable enthusiasm among academics, activists and policymakers. By creating a deliberative forum composed of randomly selected citizens, they act as a microcosm of wider society. Some democratic theorists have suggested using citizens' assemblies as sources of trustworthy, non‐partisan information about politics for the broader public. Others have gone as far as proposing them as an alternative for legislative bodies, in a form of lottocratic government [5, 6]. However, their primary function is typically thought to be to allow citizens to feed into policymaking processes, and this is also how the vast majority of them have been used. This function as policymaking tools is also the exclusive focus of this paper.

Citizens' assemblies come in many institutional forms. They can vary in duration, format and number of participants, including variations such as Citizens' Juries and Consensus Conferences [7]. Their role can range from merely advisory, providing recommendations to policymakers, to being empowered with decision‐making power over policy outcomes. Citizens' assemblies can either be institutionalised, as seen in the Citizens Council of the UK's National Institute for Health and Care Excellence (NICE), which provides ongoing input into health policy, or ad hoc and convened temporarily to address a specific issue.

Much of the critical debate surrounding citizens' assemblies has focused on issues of institutional design. For example, some concerns relate to the use of random selection: it is difficult for citizens' assemblies to include enough participants to achieve a representative sample of the population, and there are predictable biases in participation rates across different demographic groups [8]. Other concerns relate to the way their design can be manipulated by sponsors, for example, to frame deliberation in ways to suit narrow political or economic agendas^2^ [9]. Because these practical objections are well rehearsed in the literature, we will set them aside in this paper.

Instead, we will focus on scrutinising the normative foundations of citizens' assemblies.^3^ It is common to defend the use of citizens' assemblies by emphasising their potential to increase the democratic credentials and quality of policy decisions [10]. However, these ideas are seldom fully defended from first principles. By reconstructing various possible interpretations of the central values and principles that underpin the use of citizens' assemblies, we will show that their justification is not straightforward, and, indeed, perhaps more limited than often acknowledged.

## 3 Accountability for Reasonableness

A first possible argument for using citizens' assemblies begins from Norman Daniels' insight that healthcare policymaking involves questions of political legitimacy, as well as justice. Although Daniels is best known for his influential account of health justice, his engagement with real‐life controversies surrounding healthcare resource allocation led him to believe that any theory of justice is too general and indeterminate to resolve these disputes [11]. If justice is indeterminate, reasonable people may disagree about a given allocation decision. What matters, then, is that those who are disadvantaged by the decision can accept the moral authority of the decision‐makers—in other words, that the decision is *legitimate*, as well as fair.

This insight led Daniels (together with James Sabin) to develop ‘accountability for reasonableness’ (A4R), which has become arguably ‘the dominant paradigm in the field of health policy’ [12]. This framework aims to ensure legitimacy by requiring that the reasons for allocation decisions are publicly available (publicity condition), that these reasons can be recognised as relevant by fair‐minded people (relevance condition), that there are mechanisms for challenging decisions (revision and appeals condition) and that regulations ensure that these conditions are met (regulative condition) [13, 14]. Although developed initially in the context of healthcare allocation decisions, the framework can plausibly be used for a wide range of health policy questions.

How does A4R support the case for using citizens' assemblies? In fact, public or stakeholder participation is not an integral part of the framework, and it is neither necessary nor sufficient for achieving legitimacy [15, 16, 17]. On Daniels' view, public participants cannot directly contribute to legitimacy by representing broader social groups because they are not elected by constituencies, nor can they be recalled. However, they can instrumentally enhance legitimacy by increasing the likelihood that ‘a broader range of relevant reasons and rationales will be aired in the decision‐making process’ as well as by ‘providing a clear mechanism to achieve transparency’ [18]. By increasing the visibility of decision‐making (publicity condition) and widening the pool of reasons that inform it (relevance condition), citizens' assemblies can thus indirectly help secure A4R.

However, Daniels' legitimacy‐based argument provides only weak or contingent reasons for using citizens' assemblies. It will not always be the case that deliberation among randomly sampled citizens is the best way to promote either relevance or publicity. Consider first publicity. Whether or not a given citizens' assembly actually helps realise the publicity condition depends on contingent features of the wider political environment. And, in fact, while some citizens' assemblies have indeed achieved a high level of prominence in the public eye, this remains the exception. Most citizens' assemblies are not widely known among ordinary citizens and receive little media coverage [19, 20, 21].

We also have fairly weak reasons to think that using citizens' assemblies will tend to advance the relevance condition better than deliberation among experts. Consider, for example, a process aimed at reviewing antimicrobial stewardship protocols in hospitals. Although there may be circumstances where randomly selected citizens may generate relevant reasons (as we discuss in the following section), here, it seems likely that experts would be better placed to articulate complex arguments, for instance, in relation to the ethics of statistical risks and temporal discounting of future harms of bacterial resistance. At the very least, lay citizens would require a high degree of training to assess those reasons for and against different policies. Cases like this suggest that we have only weak reasons for thinking that citizens' assemblies will generally tend to be better than experts at creating a wide pool of relevant reasons to inform healthcare policy choices.

Relatedly, citizens' assemblies will not always be the most efficient way to advance A4R. Like with any decision‐making mechanism, the benefits of citizens' assemblies must be weighed against the resource and time constraints involved. For example, consensus conferences, in which 10–25 citizens selected through stratified random sampling meet over several weekends and provide recommendations to parliamentarians, cost between £30,000 and £100,000 [22]. Is this an efficient use of resources? While we may broaden the pool of relevant reasons with a citizens' assembly, increasing breadth offers diminishing returns to legitimacy: once enough relevant reasons have been publicly aired (and mechanisms for appeals have been offered), those disadvantaged by a policy would seem to have sufficient reason to accept its authority.^4^

Finally, the A4R framework appears grounded in reasoning about the value of citizen participation that presumably some citizens would reject. For Daniels, that value is conditional on the indeterminacy of justice with respect to the issue at hand. If it were possible that members of a citizens' assembly could improve the justice of the decision, then their participation would seem inappropriate. After all, when it is clear and unambiguous what justice requires, deliberation is not only unnecessary but could conceivably even lead to worse decisions. In more concrete terms: officials should invite citizen engagement only when they know that it cannot improve outcomes in this sense^5^ [23].

Yet, this way of understanding the value of citizens' assemblies seems to miss a central reason that we might have for valuing them. As we discuss in the following section, it is often thought that diverse citizen voices can substantively improve health policies, including by making them more just. Citizens' assemblies are sometimes convened primarily for the purposes of publicity and without any expectation that they might substantively improve policies. However, participants and members of the wider public often perceive this as manipulative or disrespectful, in part because of the mismatch between official claims about the value of public participation and the highly restrictive terms on which opportunities to participate are offered [24, 25]. Perhaps it is not, in fact, disrespectful to ask citizens to deliberate among equally just policies, assuming that this task is transparently communicated to them. Even so, the rationale for citizen participation grounded in A4R seems to miss one of the most prominent reasons commonly advanced in favour of citizens' assemblies. So, even though Daniels is often associated with transferring decisional powers away from professionals and towards wider publics [26], the legitimacy argument expressed in A4R seems somewhat incomplete as a normative foundation for citizens' assemblies.

## 4 The Epistemic Case for Citizens' Assemblies

An alternative argument for citizens' assemblies focuses on their ability to generate just decisions, rather than legitimate ones. The key insight of this argument, which has been articulated in debates about the epistemic value of democracy [27], is that experts and professionals tend to think alike. They are by definition more educated and informed about health policy than the wider population and also likely to be demographically distinctive in other ways, such as in relation to sex, race and (almost inevitably) social class. These similarities may incline them to groupthink or epistemic blind spots. A citizens' assembly whose participants are randomly selected could make space for reason‐giving by ‘outsiders’ with very different ways of understanding an issue, widening the pool of reasons and arguments that inform a debate. Unlike in A4R, where appealing to relevant reasons is important to ensure that those disadvantaged by a decision can accept it as legitimate, a justice‐based rationale for citizens’ assemblies would emphasise the way diverse reasons can improve outcomes, in one of two ways:

*Lived Experience:* Compared with all experts, some outsiders may have different lived experience of healthcare or health policy, which inclines them to articulate distinctive kinds of reasons in deliberation. For instance, a citizens' assembly may include some less educated or socially disadvantaged participants. Members of these groups can contribute distinctive experiences of certain aspects of healthcare provision, such as direct experiences of stigma and discrimination. Including diverse voices may also correct testimonial injustices, such as those suffered by people afflicted by illness or disability [28].
*Deliberative Wildcards:* Outsiders may often articulate lower quality arguments and reasons than experts. However, sometimes, these could push deliberation in unexpected directions, generating new kinds of high‐quality public reasons in response^6^ [29, 30]. Imagine, for instance, a situation in which a vocal minority in a citizens' assembly repeatedly insist that they should be allowed to take limousines to their dialysis appointments. To meet them halfway, officials agree to consider ordinary taxis as an alternative and later realise that this would be practicable and save money over a *status quo* that only allows the use of official ambulances.

Whether framed in terms of lived experience or deliberative wildcards or both, this kind of argument appears to establish a prima facie case for using citizens' assemblies in health policy: by closing epistemic blind spots and breaking through groupthink, citizens' assemblies can improve the epistemic quality of deliberation and help ensure that the most just health policy is selected.

Yet, it is important to recognise that this argument establishes only the *possibility*, rather than the necessity or even the probability of citizens' assemblies making better policies than experts alone. While outsiders may have greater capacity to break through policy groupthink, they may also sometimes make bad decisions due to their lack of normative expertise. In Oregon, in the late 1980s, for example, a public consultation was held to decide which procedures would be covered by its Medicaid programme. Citizens in this consultation decided to give priority to vasectomies over hip replacements, even though the latter are fundamental for mobility, pain relief and social participation [31]. This is not to say that the benefits of expertise will always trump the risks of groupthink. The point, rather, is that it is not possible to decide *a priori* which consideration is more decisive—let alone whether inserting citizens' assemblies into decision‐making processes would tend to lead to epistemic improvements.^7^ It would require empirical evidence to show that citizens' assemblies reliably tend to improve outcomes, but this evidence does not currently exist^8^ [32, 33].

It may be replied that officials could simply ignore poor suggestions by citizens' assemblies, if the latter are designed to play a merely consultative role. However, in order for citizens' assemblies to have desirable epistemic effects, there would need to be norms of deference that push officials to genuinely consider epistemic wildcards and lived experiences. With those norms in place, there would also be at least some pressure to accept poor suggestions.

Even if citizens' assemblies can sometimes improve the substantive justice of a health policy decision, they might create undesirable kinds of policy inconsistency. Consistent policy over time is valuable to satisfy formal requirements of justice—treating like cases alike—and it also facilitates planning and co‐ordination amongst citizens^9^ [34]. Yet, health policy made by citizens' assemblies is less likely to be consistent with earlier policy than if it were made by experts. The latter have more familiarity with precedent and with the kinds of policy reasoning and learning processes that underpin existing policies.

Finally, it is not clear that we need citizens' assemblies *specifically* to create most or all the epistemic benefits associated with inputs from ‘outsiders.’ To understand citizens’ lived experiences, officials could consult polls and surveys, interview groups of patients and service users, consult with civil society groups representing people with specific medical conditions and so on. In fact, focusing on the experiences of stakeholders and affected parties would intuitively yield greater epistemic benefits than consulting a random sample of the population for many health policy questions.^10^

Perhaps wildcards could be generated by soliciting online or offline feedback on policy proposals and providing public replies, or by tasking other officials, experts or AI systems with challenging policy proposals robustly, aiming to play devil's advocate, identify weak points and develop the best possible counterarguments, including ones that look *prima facie* outlandish. These approaches may be more time‐ and cost‐efficient ways to elicit wildcards and, if tailored specifically for this purpose, also more effective than a process that only occasionally would throw out wildcards organically in the course of deliberation.

A defender of citizens' assemblies could argue, however, that wildcard ideas would need to be aired in public for officials to even consider taking them seriously. Because they involve reasons that are not intrinsically high quality, bureaucrats may be tempted to discount them if they are conjured up by an AI system, for instance. With a citizens' assembly, by contrast, officials may face discontent or reputational damage if they discount them like this, so wildcards really could push deliberation in new, productive directions.

While this objection is fairly plausible, it does not epistemically vindicate citizens' assemblies. Any institution that creates reputational pressure for officials to take wildcards seriously will also create similar pressures for them to take other bad ideas and arguments seriously. Overall, therefore, it is not clear whether the epistemic gains from attending to wildcards will outweigh the losses from pressure to make policy on the basis of bad reasons.

The epistemic argument, unlike A4R, does provide us with a clear reason for using citizens' assemblies to make healthcare policy. Yet, it is not at all clear how weighty that argument is. To gain insight into that question, we would need empirical evidence about the relative epistemic advantages of outsiderness relative to expertise and about the ‘value added’ of citizens' assemblies relative to other ways of eliciting input from outsiders. There is scope for much more research on both topics.

## 5 A Deliberative Democratic Argument

A final argument draws on deliberative democratic ideas about the way citizens' assemblies can help with a much‐needed ‘“democratization” of the administration.’ [35] This argument begins with the observation that the traditional source of legitimacy in democratic societies is the representative process: laws and policies ideally are determined through parliamentary debate and decision‐making, and then interpreted and implemented by the bureaucracy. In practice, however, this model is under strain. The sheer volume and complexity of contemporary policy decisions mean that many or most can no longer plausibly occur directly in legislatures. Hence, in practice, it is the bureaucracy that does most policy work. Some of this extra‐parliamentary decision‐making involves moral questions on which there is no consensus, and in such cases, citizens' assemblies may bolster the legitimacy of the process or the resulting decisions.

Unlike in A4R, legitimacy here does not depend only on a regulated process that articulates relevant public reasons. Instead, the argument assumes that legitimacy is generated in multiple ways: *output legitimacy* derives from the substantive quality of decisions, *input legitimacy* from the inclusive nature of participation in making them and *throughput legitimacy* from the qualities of the decision‐making process itself, such as its expression of values of equal respect^11^ [36, 37].

Citizens' assemblies may advance output legitimacy through the epistemic benefits associated with decision‐making with or by a diverse group of citizens. Having already argued that these benefits are uncertain, let us briefly consider the other two dimensions of legitimacy. First, citizens' assemblies may improve input legitimacy by promoting democratic participation among ordinary citizens [38]. While plausible in principle, a clear limit to this argument is the small size of citizens' assemblies: only a few dozen or hundred participants get to participate. If encouraging participation is the goal, there are plausibly more effective instruments that can reach a greater share of the population, ranging from civic education to measures aimed at strengthening civil society.

What about the argument that citizens' assemblies may support throughput legitimacy because they symbolically express respect? Creating spaces for officials to listen to the voices of ordinary citizens may well have this effect, so long as the act of listening is not merely performative or strategic. Yet even if officials are genuinely open to citizen input, citizens' assemblies involve a relatively top‐down form of listening that happens within parameters narrowly defined by officials and their concerns [39]. Other things being equal, decision‐making on equal terms seems more respectful, which suggests empowering citizens' assemblies, rather than merely giving them a consultative role.

Yet, we have good reasons for leaving the last word on policy questions to elected officials, rather than participants in citizens' assemblies, where possible. The former have much greater incentives for responsiveness and accountability to the citizenry than the latter [40, 41]. They also have powerful reasons to consider the trade‐offs between policy proposals across all policy domains and to develop a coherent bundle of policies on this basis [42]. Citizens' assemblies, by contrast, must have a relatively narrow focus to arrive at policy recommendations in a limited time. Decision‐making by the elected will usually generate a livelier sense of collective identification with policies, because parties have electoral incentives to educate the public about their moral basis [43]. Finally, making sustainable policy is not simply an epistemic exercise but also a practical matter of assembling a coalition that will support policy and work to maintain it over time—and parties are the obvious agents of such coalitional bridge‐building^12^ [44].

As already noted, however, the volume and complexity of policy decisions mean that they cannot all be made by elected officials. When, then, should we deviate from this default option and instead delegate policy to citizens' assemblies instead? The logic of the deliberative argument about the need to democratise the administration points towards ‘big’ questions (for instance, about the broad outlines of health policy) being reserved for parliaments. At the same time, very ‘small’ questions should best be handled by bureaucrats directly, because delegating them to a citizens' assembly would face the same issues of cost‐effectiveness that we discussed in the context of Daniels's theory. The *goldilocks* principle seems to apply: issues for citizens' assemblies must not be too big nor too small. However, how to draw the boundaries is an open question.^13^

In sum, while the deliberative democratic argument provides perhaps the strongest case for citizens' assemblies, it is also highly constrained. It presents citizens' assemblies as useful because they offer remedial legitimacy in the face of strained representative institutions. Yet, even on these terms, their contributions are limited. Their epistemic benefits are uncertain. Their potential to increase participation is limited by their small scale. And their ability to express respect is most plausible when they are empowered, which makes sense only for an indeterminate range of mid‐level policy contexts where our default representative institutions are ill‐suited to the task.

## 6 Concluding Thoughts

In this paper, we have attempted to reconstruct the strongest rationales for using citizens' assemblies to make health policy: Daniels's influential A4R framework; a justice‐based argument focusing on the epistemic quality of decisions; and a deliberative democratic argument that incorporates considerations about input, throughput and output legitimacy for decisions.

The A4R argument for deliberative citizens' assemblies is contingent or weak. It is not clear that involving ordinary citizens is always a good way to advance publicity or relevance. Moreover, because A4R is reserved for decisions where justice is indeterminate, it seems to miss what is commonly considered one of the key reasons to employ citizens' assemblies, namely, their potential to substantively improve the justice of policy outcomes.

The epistemic argument articulates one such reason for valuing citizens' assemblies, one that turns on their ability to break through groupthink and close epistemic blind spots. However, the weight of that reason is far from clear. We are currently lacking much sense of the concrete value of outsiderness relative to expertise in health policy processes. Even if we are persuaded that health policy is currently far too reliant on experts, citizens' assemblies may not be the best way of eliciting input from outsiders, compared to feasible alternatives.

The deliberative argument for using citizens' assemblies is stronger than the others. Or, more strictly, its claims about the potential for generating input and throughput legitimacy for decisions are prima facie persuasive. Yet, the weight of these benefits may be limited by citizens' assemblies' small size and their merely consultative status. More research is needed to establish when input and throughput benefits really are weighty enough to support the use of citizens' assemblies, including by refining the *goldilocks principle* that citizens' assemblies, if empowered, should take decisions that are neither too ‘big’ nor too ‘small.’

Of course, these arguments identify certain conditions in which using citizens' assemblies may be beneficial: if we think them very likely to advance publicity; or generate good epistemic reasons, for instance, because the bureaucracy is not diverse and we have specific reasons to prefer citizens' assemblies to alternative methods for eliciting inputs from outsiders; or improve input and throughput legitimacy for ‘goldilocks‐style’ policy questions.

While the arguments considered provide some justifications for making health policy with citizens' assemblies, those justifications are not obviously weighty or generally applicable enough to explain the substantial recent enthusiasm for these events. It may be that the officials who create them do so for reasons not generally considered by political theorists and philosophers. We therefore conclude by speculating about two possible empirical interpretations of the movement towards citizens’ assemblies.

According to a relatively *cynical interpretation* that generalises from some clearly manipulative citizens' assemblies [45], officials, encouraged by an army of public participation professionals [46], may institute citizens' assemblies to create empirical legitimacy in a context of widespread political distrust. States have come to intervene into more and more aspects of our lives [47] and to demand increasing amounts of tax from us [48], even as more educated and affluent citizens have become more critical of political authorities and authority in general [49]. This exerts pressure on officials of almost all kinds to be visibly responsive to the communities subject to their decisions. Citizens' assemblies can make decisions look inclusive and democratic. Officials might value this empirical legitimacy even at the expense of normative legitimacy or justice, including because it could help them fight internal battles with other officials by making their priorities look widely valued.

An alternative analysis of the rise of citizens' assemblies could accept the realist insight that they are adopted for strategic reasons but argue that they have sometimes been used as *weapons of the weak*. Some of the academics, activists and officials who promote citizens' assemblies might reasonably see them as an alternative to default policy pathways that are defective because they run through corrupt or dysfunctional parties, legislatures and bureaucracies. If legislators tend to be in the pockets of big pharmaceutical companies, for instance, it may well make sense for bureaucrats to create a citizens' assembly as a space for new voices and to increase the reputational costs for corrupt or dysfunctional actors. The Irish Constitutional Convention, for instance, helped legalise abortion, which was a very popular reform to a policy that both major parties had been previously unwilling to take action on [50]. This interpretation of the spread of citizens' assemblies might be especially useful for understanding their appeal in the modal political system, worldwide, whose parties and bureaucrats are relatively corrupt or dysfunctional.

Of course, the realism of this analysis cuts both ways: the powerful can use citizens' assemblies themselves in corrupt or dysfunctional ways [51, 52]. On this realist account, citizens' assemblies' value ultimately depends on the highly situation‐specific ways in which they combat or reinforce injustice. It is an empirical question which way they tend to tilt in practice. However, as we have suggested, there is nothing inherent in the institutional design of citizens' assemblies that makes them a particularly promising weapon of the weak.

Whatever the empirical reasons for the current enthusiasm for citizens' assemblies in health policy circles, we have provided some reasons for caution about the strength and general applicability of some of the most obvious kinds of arguments in favour of using citizens' assemblies to promote justice and normative legitimacy. The best rationales for using citizens' assemblies for health policy give us reason to value them only in a specific range of circumstances whose exact nature and boundaries require much more research.

## Conflicts of Interest

The authors declare no conflicts of interest.

## Endnotes

1 We use the term ‘citizens' assemblies’ in a generic way to refer to an entire class of institutions that involve citizen deliberation about policy (see Section 2, below). Political theorists often use the term ‘deliberative minipublic’ in this generic way, reserving “Citizens' Assembly” for a specific institutional design. Yet citizens' assembly has more resonance outside of narrow academic circles than minipublic.

2 For instance, Cadbury Schweppes sponsored a series of minipublics on childhood obesity that framed the issue in terms of personal responsibility and took off the table sensible regulative options like mandatory warning labels on unhealthy food.

3 Unless otherwise noted, our discussion applies to citizens' assemblies of all types (consultative or empowered, ongoing or ad‐hoc).

4 This, at least, is how we believe Daniels might understand the relation between the breadth and relevance of reasons. It is possible to reinterpret the relevance condition so as to refer not to *breadth*, but *certainty* about the relevance of reasons. In other words, citizens' assemblies could be justified not because they generate diverse reasons, but because they help establish that the reasons generated are relevant. Resources might then be justified on account of their contribution to certainty. However, there is no reason to think that lay citizens are better positioned to ascertain the relevance of reasons any more than experts: our arguments above about their ability to generate relevant reasons without a high degree of training would also apply, ceteris paribus, to their ability to ascertain their relevance.

5 Although Daniels sometimes gestures towards the possibility that A4R might increase the substantive justice of outcomes, this idea is incompatible with the foundational conceptual and normative commitments of A4R, which is designed to resolve questions on which there is reasonable disagreement, implying a range of outcomes, none of which is preferable from the perspective of justice.

6 This argument mirrors Hélène Landemore's ‘diversity trumps ability’ argument when interpreted—as she tells her readers it should be—as one that draws directly on the agent‐based modelling of Lu Hong and Scott Page. Some of her presentations of the argument, such as in the discussion of ‘Twelve Angry Men,’ instead more closely resemble arguments from lived experience.

7 A defender of citizens' assemblies could argue that the trade‐off identified here between expertise and ‘outsiderness’ could be mitigated in a well‐designed citizens' assembly through education and training that reduces the likelihood of very bad decision‐making without undermining participants’ abilities to avoid groupthink and blind spots. Their ability to function as wildcards would presumably not survive education and training, but perhaps they could still make good use of their lived experience in the decision‐making process. This argument, while reasonable, does not show that there is never a trade‐off between expertise and outsiderness. Nor does it show that health policy is currently too reliant on experts and likely to be improved by granting more of a role to outsiders in citizens' assemblies.

8 In lieu of empirical evidence, Landemore tries to ground the claim that citizens' assemblies tend to make better decisions than experts with an analogy between real policy decisions and a series of simulations in which more cognitively diverse agents outperformed more individually competent agents at problem‐solving tasks (see Hong and Page, “Groups of diverse problem solvers can outperform groups of high‐ability problem solvers”). Yet, it does not seem likely that the results of this simulation can be extrapolated to the real world, not least because the simulations assume that agents do not disagree at all on matters of value.

9 Daniels strongly values consistency in health policy and this partly explains his commitment to publicity. He argues that a public record of health policy decisions and their justifications could encourage consistency in much the way that a body of case law does: by serving as a repository of information about how situations were evaluated and responded to, thereby setting precedents for future health policy decisions.

10 Daniel Hausman appears to have identified one very good reason to favour citizens’ assemblies over such alternatives in processes of evaluating the value of health states. See his contribution to this special issue.

11 These three kinds of legitimacy correspond to the three functions sometimes attributed to deliberative systems: the ‘epistemic’ function of arriving at wise decisions (which would generate output legitimacy); the ‘democratic’ function of making decisions in an inclusive way (input legitimacy); and the ‘ethical’ function of expressing respect (throughput legitimacy).

12 Variation in European welfare expenditures in the latter half of the twentieth century mostly depended on the nature and inclusiveness of the cross‐class coalition that first established and then supported the welfare state. Obamacare is a prominent example of a health policy decision that created a constituency of beneficiaries whose support then made it difficult to reverse.

13 The deliberative democratic argument may give non‐state organisations—such as international health NGOs or insurers—especially good reasons to set up citizens' assemblies if they lack ‘first best’ elective offices and it would be difficult to establish such offices.
